# GLP-2 Prevents Intestinal Mucosal Atrophy and Improves Tissue Antioxidant Capacity in a Mouse Model of Total Parenteral Nutrition

**DOI:** 10.3390/nu8010033

**Published:** 2016-01-09

**Authors:** Qiucheng Lei, Jingcheng Bi, Xinying Wang, Tingting Jiang, Chao Wu, Feng Tian, Xuejin Gao, Xiao Wan, Huijun Zheng

**Affiliations:** 1 Department of General Surgery, Jinling Hospital Affiliated to Southern Medical University, Nanjing 210002, China; lqiuchenggd@163.com (Q.L.); xuejingao870214@163.com (X.G.); 2 Research Institute of General Surgery, Jinling Hospital, School of Medicine, Nanjing University, Nanjing 210002, China; ahbijingcheng@163.com (J.B.); jiangtingting08med@163.com (T.J.); wuchao0008@126.com (C.W.); easyhard666@163.com (F.T.); van395412495@sina.cn (X.W.); huijunzheng@163.com (H.Z.)

**Keywords:** glucagon-like peptide-2, antioxidant capacity, proliferation, apoptosis, GRP78

## Abstract

We investigated the effects of exogenous glucagon-like peptide-2 (GLP-2) on mucosal atrophy and intestinal antioxidant capacity in a mouse model of total parenteral nutrition (TPN). Male mice (6–8 weeks old) were divided into three groups (*n* = 8 for each group): a control group fed a standard laboratory chow diet, and experimental TPN (received standard TPN solution) and TPN + GLP-2 groups (received TPN supplemented with 60 µg/day of GLP-2 for 5 days). Mice in the TPN group had lower body weight and reduced intestinal length, villus height, and crypt depth compared to the control group (all *p* < 0.05). GLP-2 supplementation increased all parameters compared to TPN only (all *p* < 0.05). Intestinal total superoxide dismutase activity and reduced-glutathione level in the TPN + GLP-2 group were also higher relative to the TPN group (all *p* < 0.05). GLP-2 administration significantly upregulated proliferating cell nuclear antigen expression and increased glucose-regulated protein (GRP78) abundance. Compared with the control and TPN + GLP-2 groups, intestinal cleaved caspase-3 was increased in the TPN group (all *p* < 0.05). This study shows GLP-2 reduces TPN-associated intestinal atrophy and improves tissue antioxidant capacity. This effect may be dependent on enhanced epithelial cell proliferation, reduced apoptosis, and upregulated GRP78 expression.

## 1. Introduction

Total parenteral nutrition (TPN) is a critical therapeutic modality for patients with impaired gut function, such as patients with short bowel syndrome, severe inflammatory bowel disease, or chronic idiopathic intestinal pseudo-obstruction [1,2,3]. According to the Mayo Health System report in 2006, there were more than 1000 infants requiring TPN every year [4]. In recent years, almost 400,000 patients per year in the United States were dependent on TPN or intravenous feeding care for survival [5]. Despite its clinical importance, long-term TPN can lead to reduced epithelial cell (EC) proliferation, increased EC apoptosis and mucosal atrophy [6,7], all of which may contribute to an associated increase in risk of infectious complications [8,9]. Additionally, TPN-associated intestinal hypoplasia and epithelial cell injury were strongly linked to gut barrier dysfunction [10] and increased bacterial translocation [11]. However, the potential mechanisms contributing to TPN-associated intestinal atrophy and epithelial cell injury remain poorly understood. It has recently been shown that intestinal growth factors contribute to epithelial repair [12], performing functions such as promotion of cell proliferation and inhibition of apoptosis, making it an attractive target for prevention of intestinal damage during TPN administration.

Glucagon-like peptide-2 (GLP-2), an intestinal growth-promoting factor, is a 33-amino acid peptide derived from the enteroendocrine L cells of the intestinal epithelium [13], which directly or indirectly stimulates intestinal nutrient absorption and growth [14,15]. Exogenous GLP-2 administration also exerts profound effects on stimulating intestinal cell survival and proliferation [16]. Other biological roles ascribed to GLP-2 are increased intestinal blood flow [17], anti-inflammatory effects [18], and reduced gut permeability [19]. Recently, it was demonstrated that exogenous GLP-2 possesses antioxidant properties, which protect against intestinal injury [20]. Arda-Pirincci *et al.* found that subcutaneous treatment with a GLP-2 analogue reversed morphological damage, oxidative stress, and cell apoptosis by markedly reducing lipid peroxidation in experimental mice with lung injuries [21]. Similar results were observed in another study, which revealed GLP-2 treatment could decrease oxidative damage in a rat model of cerebral ischemia/reperfusion [22]. However, so far, there have been no studies to date on the effect of GLP-2 on antioxidant activity in a TPN model, and the mechanisms of this property have yet to be elucidated.

There is a substantial body of evidence that TPN causes intestinal atrophy, loss of epithelial cells, and an increase in mucus damage [6,7]. Additionally, the reduction in antioxidant capacity during TPN administration is a known phenomenon that already been demonstrated in several experimental and clinical studies, which indicated increased free radical attack and reduced glutathione under TPN [23,24,25]. Interestingly, intestinal atrophy induced by TPN is strongly related to the loss of GLP-2 secretion [16,26]. Thus, based on these effects of GLP-2, we hypothesized that exogenous GLP-2 may protect against TPN-associated intestinal atrophy and increase intestinal antioxidant capacity.

Proliferating cell nuclear antigen (PCNA), an important regulator of the cell cycle, is a 36-kDa molecule that acts as a marker of proliferative activity in different cells [27,28]. Conversely, the cleaved caspase-3 protein is part of a family of cysteine proteases, and acts as an effector of apoptosis [29]. Additionally, the 78-kDa glucose-regulated protein (GRP78) is one member of an endoplasmic reticulum chaperone family, which regulates protein folding and Ca^2+^ balance in the endoplasmic reticulum [30]. It has been suggested that GRP78 not only improves cell survival and accelerates cell proliferation, but also protects cells from oxidative damage [31,32]. We therefore hypothesized that there would be improvements in both gut atrophy and an increase in antioxidant levels in response to GLP-2 treatment, which could be associated with increased PCNA and GRP78 secretion, and reduce cleaved caspase-3 expression in the intestine.

## 2. Materials and Methods

### 2.1. Animals

This study was approved by the Animal Care and Use Committee of Jinling Hospital, Nanjing, China. Male, specific pathogen-free six to eight weeks old Institute of Cancer Research mice (the Laboratory Animal Research Center of Jiangsu University, Zhenjiang, China) were housed on a 12:12-h light/dark cycle under controlled temperature and humidity conditions. The mice had free access to a standard laboratory diet prior to the study, and were unrestricted in activity. They were allowed to acclimatize for five days before the study protocol.

### 2.2. Operative Procedure, TPN Administration, and Collection of Tissue Sample

A total of 24 male mice weighing 30–35 g each were randomly divided into three groups (for each group *n* = 8): A control group received a diet of standard laboratory chow, and two experimental groups received continuous infusion of TPN, or TPN plus a synthetic recombinant analogue of human GLP-2 (Teduglutide, purity >90%, PLLabs, Canada). Mice were anesthetized with an intraperitoneal injection of ketamine (100 mg/kg body weight) and surgically implanted with rubber catheters (0.305 mm inner diameter, 0.635 mm outer diameter; Helix Medical Inc., Carpentaria, CA, USA) in the jugular vein, and adapted to their diet treatment within 24 h post surgery, as reported previously [33]. Meanwhile, all mice received 0.9% saline through the catheters for 48 h during surgical recovery. After two days, mice in the TPN and TPN + GLP-2 treated groups received optional water and featured fluid intake of TPN at 4.4 mL/day on the first day, 7.7 mL/day on the secondly day, and 11 mL/day for the final three days of the experimental period [33]. The mice in the control group were infused intravenously with 0.9% saline via the jugular vein, and had free access to standard laboratory chow and water. The GLP-2 was dissolved in sterile phosphate buffered saline (PBS), and mice were injected via the rubber catheter twice daily for five days with 30 µg of GLP-2 (a total of 100 µL solutions). Controls were injected with the vehicle (100 µL PBS). The TPN solution consisted of 5.3% free amino acids, 32% dextrose, and multivitamins and electrolytes (a total of 1280kcal/L with a non-protein calories/nitrogen rate of 149:1) [33]. The weight of each of the mice in all groups was measured daily using an electronic balance at the beginning of experiment (initial) and on day 7 (final) (two days for surgical recovery plus five days for the experimental period). After five days of feeding each group (*i.e.*, seven days catheterization), mice were weighed and terminally anesthetized as described above, and whole small intestines were removed. The lengths of the small intestines were measured. When measuring the length, two segments of the small intestine, approximately 0.5 cm in length, were used for morphological analysis, and the remaining tissue was split into small sections of approximately 0.5–1.0 cm and subjected to molecular analysis. All tissues were stored at −80 °C, except the tissues used in the morphological analysis that were soaked in 4% paraformaldehyde and stained at 4 °C.

### 2.3. Morphology

#### 2.3.1. Light Microscopy

Segments of small intestine of approximately 0.5 cm in length, taken from approximately 2 cm from the end of ileum were opened along the mesenteric border and fixed in 10% neutral buffered formalin. After dehydration and wax embedding, these tissue samples were stained with hematoxylin and eosin (H & E), following standard protocols. Villus length and crypt depth of the mice in three groups (*n* = 8 mice per group) were measured in the coded sections (10 intact villus-crypt units per section, and at least two sections were calculated to give an average for each mouse) using a calibrated eyepiece by an investigator blinded to the sample identities. Data were analyzed with a computer-supported image analysis system (NIS-Elements AR 3.0 software; Nikon, Melville, NY, USA).

#### 2.3.2. Electron Microscopy

Another segment of small intestine of approximately 0.5 cm in length from each sample was collected and fixed with 2% glutaraldehyde in 0.1 mol/L sodium cacodylate buffer for 2 h. It was subsequently transferred to sodium cacodylate buffer and stored at 4 °C overnight. These tissues were subsequently processed for electron microscopy. NIS-Elements AR 3.0 software (Nikon, Melville, NY, USA) was used to analyze the coded photomicrographs per group (*n* = 6 mice per group) and measure microvillus length by an investigator blinded to the sample identity.

### 2.4. Intestinal Tissue Total Superoxide Dismutase (T-SOD) Activity Measurement

Intestinal tissue samples from all mice from each group were collected and immediately placed into an ice-cold RIPA lysis buffer (25 mmol/L Tris-HCl pH 7.6, 150 mmol/L NaCl, 1% NP-40, 1% sodium deoxycholate, and 0.1% sodium dodecyl sulfate containing 1 mmol/L PMSF, and 1% protease inhibitor cocktail). The samples were subsequently homogenized, and the homogenate was then centrifuged at 3500 rpm/min for 10 min. The Total Superoxide Dismutase Detection Kit (A001-1; Nanjing Jiancheng Bioengineering Institute, Nanjing, China) was used to analyze the T-SOD activity, using the xanthine oxidase method based on the production of O_2_^−^ anions. The generation of nicotinamide adenine dinucleotide phosphate was assayed spectrophotometrically by measuring absorbance at 340 nm, and T-SOD activity was expressed as units per milligrams of protein (U/mg protein).

### 2.5. Intestinal Tissue Reduced-Glutathione (GSH) Measurement

Intestinal tissues were homogenized on ice with standard saline solution, then the homogenate was centrifuged for 10 min at 3500 rpm/min. Supernatants were transferred into fresh tubes for evaluation. The GSH levels were assessed by the Chemical Assay Kit (A006-1; Nanjing Jiancheng Bioengineering Institute, Nanjing, China). The assay was performed according to the manufacturer’s instructions, and the sample absorbance was read at 420 nm using a spectrophotometer. GSH levels were expressed as milligrams GSH per grams of protein (mgGSH/g protein).

### 2.6. Western Blot Analysis

Total concentration of proteins of small intestine tissue were determined using the BCA protein assay kit (Sangon Biotech Co., Shanghai, China), there was an equal protein concentration of 2.5 µg/µL in each sample. Equal quantities of total protein (60 µg) were electrophoresed in 10% SDS-polyacrylamide gel. The separated proteins were transferred onto a polyvinylidene fluoride membrane (Millipore Co, Billerica, MA, USA). The membranes were blocked for non-specific binding proteins in 5% non-fat milk for 1 h at room temperature and then incubated overnight at 4 °C with mouse anti-PCNA antibody (1:5000; Abcam, Cambridge, UK), goat anti-GRP78 antibody (1:1000; Santa Cruz Biotechnology, Beverly, MA, USA), rabbit anti-cleaved caspase-3 antibody (1:1000; Cell Signaling Technology, Beverly, MA, USA) and mouse anti-GAPDH antibody (1:10,000, Santa Cruz Biotechnology, Beverly, MA, USA). Subsequently, the membranes were washed three times in Tris-buffered saline with Tween-20 (9005-64-5; BBI Life Sciences Corporation, Shanghai, China) and incubated with an appropriate species-specific secondary antibody (1:10,000; Cell Signaling Technology, Beverly, MA, USA) at room temperature for 1 h. Blots were developed using chemiluminescence detection reagents and exposed to a Kodak XAR film (Eastman Kodak, New Jersey, MN, USA). All band densities were analyzed with ImageJ software, using GAPDH bands as an internal control. The results were reported in the form of density ratio of the target protein to GAPDH.

### 2.7. Real-Time Fluorescent Quantitative Polymerase Chain Reaction Analysis (Real Time Q-PCR)

The expression of small intestinal PCNA and GRP78 genes was measured using real time Q-PCR. Total RNA was extracted from intestines using the PrimeScript RT reagent Kit with cDNA (TaKaRa, MN, Japan) according to manufacturer’s protocol. The primers for the PCNA and GRP78 genes were as follows: (1) PCNA-Forward: 5′-TTTGAGGCACGCCTGATCC-3′, PCNA-Reverse: 5′-GGAGACGTGAGACGAGTCCAT-3′; (2) GRP78-Forward: 5′-CAGAGCTGTGCAGAAACTCC-3′, GRP78-Reverse: 5′-CCAACACTTTCTGGACAGGC-3′. Reaction system: 0.2 µL Rox, 5 µL SYBR solution, 0.2 µL upstream primer, 0.2 µL downstream primer and 4.4 µL cDNA. The amplification was performed using Real-time PCR system (StepOne Plus, Applied Biosystems, Waltham, MA, USA). The following time and temperature profile was used for 40 cycles: 95 °C for 30 s, 95 °C for 5 s, 65 °C for 31 s. The mRNA levels of intestinal tissue PCNA and GRP78 were measured using the 2-∆∆Ct method.

### 2.8. Statistical Analysis

Data were expressed as mean ± standard deviation (mean ± SD) and analyzed with SPSS 17.0 software (SPPS Inc. Chicago, IL, USA). The comparison of mean variability among all groups was performed by one-way ANOVA followed by the Fisher’s least significant difference *post hoc* analysis. If there were heterogeneous variances, the Dunnet’s T3 test was used. Results were considered statistically significant when the *p* value was less than 0.05.

## 3. Results

### 3.1. Body Weight

**Figure 1:**
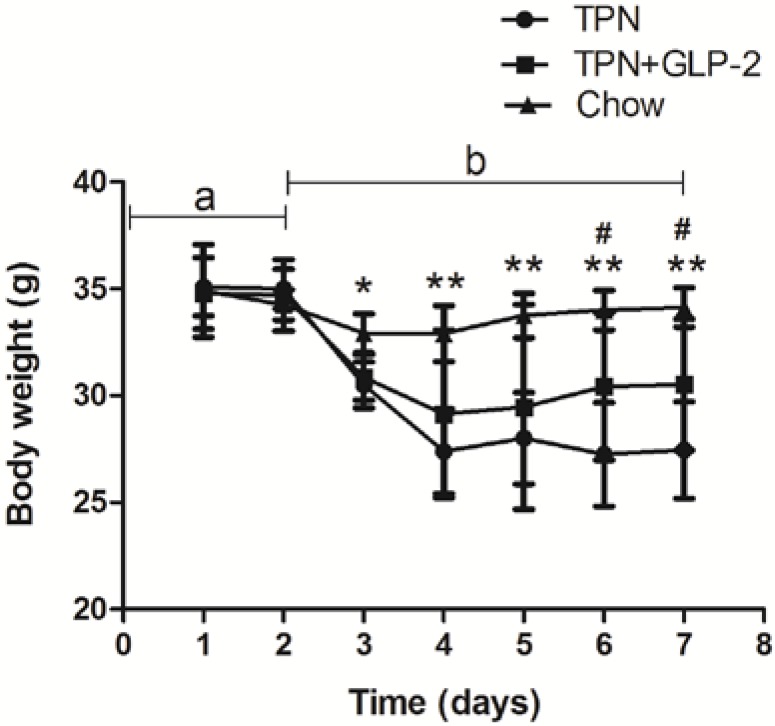
Daily body weights in all mice, a: 2-day surgical recovery, b: 5-day experimental period, * *p* < 0.05: TPN *vs.* control, ** *p* < 0.001: TPN *vs.* control, # *p* < 0.05:TPN + GLP-2 *vs*. TPN.

The mice in the three groups weighed a similar average of 33.6 ± 1.2 g at the beginning of the study. Neither body weight nor weight gain of mice was affected by TPN and GLP-2. Body weight was low in all mice in the TPN group throughout the study. On day 3, compared with the control group, body weight was lower in the TPN group (*p* = 0.002) and in the TPN + GLP-2 group (*p* > 0.05, Figure 1A). On day 6, mice in the TPN + GLP-2 group had a higher body weight compared to those in the TPN group. On day 6, mean body weight changes were −0.7 g for the control group, −6.4 g for TPN group, and −4.1 g for the TPN + GLP-2 group, respectively. The significant differences between the three groups were observed (all *p* < 0.05). On day 7, compared with day 1, weight of mice was significantly higher in the TPN + GLP-2 group than in the TPN group (*p* = 0.018).

### 3.2. Small Intestinal Length and Morphology

**Figure 2:**
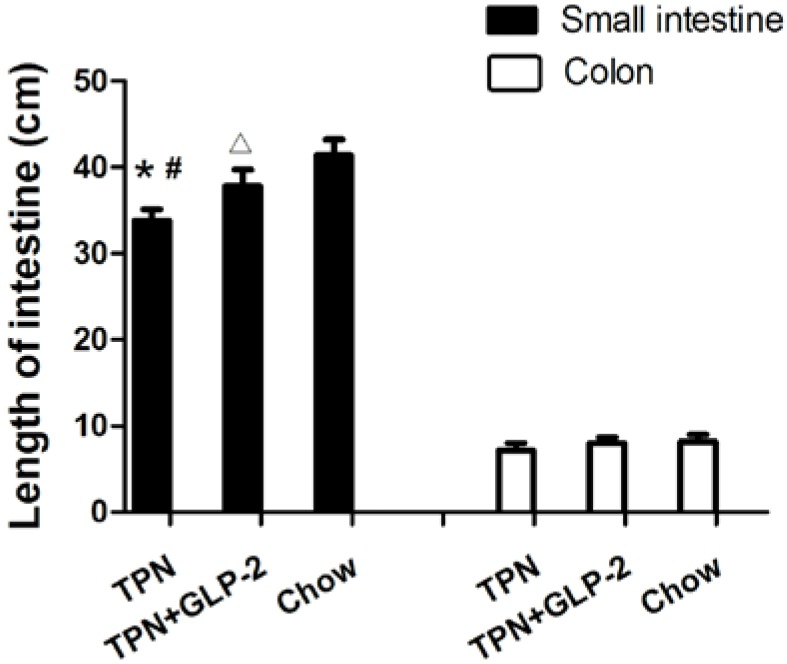
Influence of TPN administration and GLP-2 supplementation on the intestinal length. * *p* < 0.05 *vs.* TPN+GLP-2, # *p* < 0.001 *vs.* control, ∆ *p* < 0.05 *vs.* Control.

**Figure 3:**
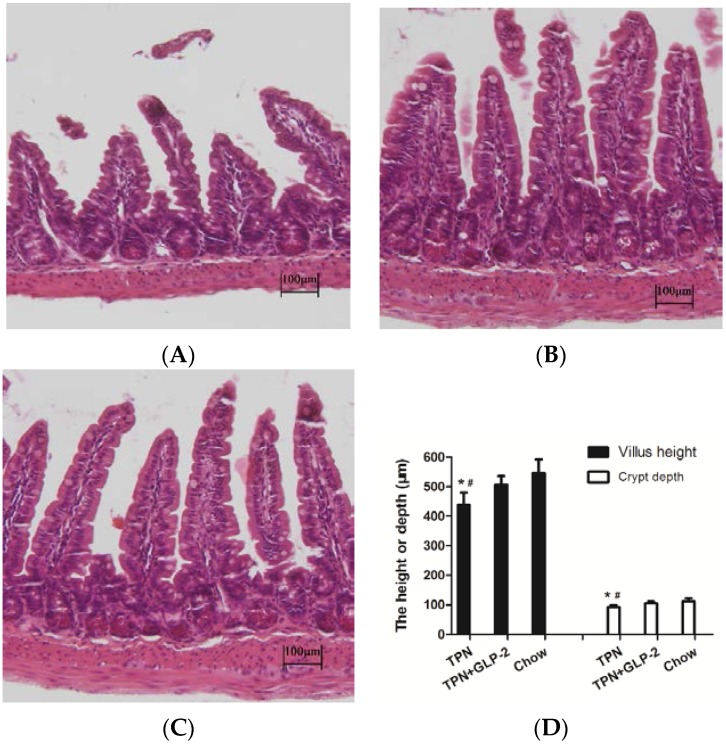
Representative H & E-stained histology image from the TPN group (**A**); TPN + GLP-2 group (**B**); and control group (**C**); (H & E staining; original magnifications, ×200) (**D**). Villus height and crypt depth in the TPN, TPN + GLP-2, and control groups are shown.* *p* < 0.05 *vs.* TPN + GLP-2, # *p* < 0.001 *vs.* Control.

**Figure 4:**
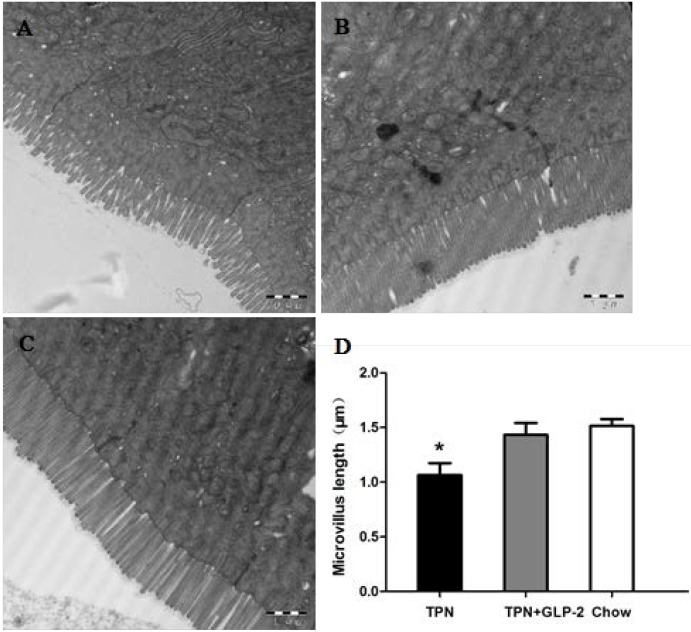
Representative photomicrographs of intestinal microvillus from the TPN group (A); TPN + GLP-2 group (**B**); and control group (**C**) (photomicrographs; original magnifications, ×20,000; bar = 1 µm). (**D**) Effect of GLP-2 treatment for five days on microvillus length (mean ± SD), * *p* < 0.05 *vs.* TPN + GLP-2 and control.

Histological assessment of the small intestine samples revealed that there was severe blunting of the villi in the TPN group. By day 7, the intestinal length in the TPN group was shorter than that in the control group (33.8 + 1.3 cm *vs.* 41.4 + 1.8 cm, *p* < 0.001) and in the TPN + GLP-2 group (33.8 + 1.3 cm *vs.* 37.8 + 1.9 cm, *p* = 0.003) (Figure 2). However, there was no significant difference in colon lengths between the three groups (all *p* > 0.05) (Figure 2). Morphometric analysis of the small intestine showed that the villus length and crypt depth were significantly lower in the TPN group compared with the control group (all *p* < 0.05). However, the GLP-2 treatment markedly improved this effect (all *p* < 0.05, Figure 3). Electron microscopy demonstrated that microvilli were longer for the TPN + GLP-2 group than for the TPN group (*p* = 0.001, Figure 4). TPN treatment changed the shape of the microvilli causing them to become shorter and less orderly, however, GLP-2 reversed this effect by altering the enterocyte morphology.

### 3.3. Changes in Intestinal Tissue T-SOD Activity and GSH Level

**Figure 5:**
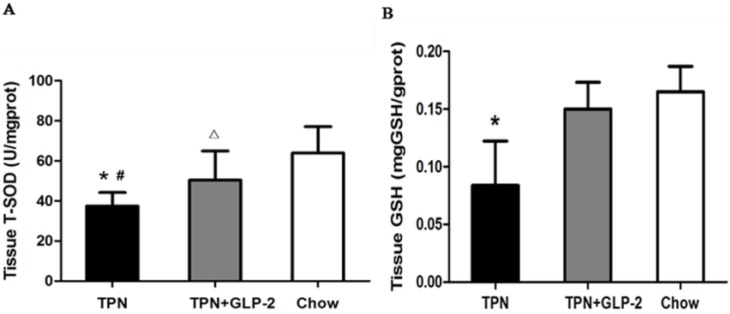
Tissue T-SOD activity and GSH level expression in mice given TPN, TPN + GLP-2 or chow for five days (mean ± SD). (**A**) T-SOD expression, * *p* < 0.05 *vs.* TPN + GLP-2, # *p* < 0.001 *vs.* control, ∆ *p* < 0.05 *vs*. control; (**B**) GSH expression, * *p* < 0.001 *vs.* TPN + GLP-2 and control. Prot: protein.

The TPN group showed oxidative damage in the intestinal tissue, as indicated by decreased T-SOD activity and GSH level. As shown in Figure 5A, T-SOD activities were significantly decreased in the TPN group compared to the control group (*p* < 0.001), and this decrease in expression was partially prevented by the addition of GLP-2 to the TPN solution (*p* = 0.042). In accord with our initial hypothesis, GSH was decreased in intestinal tissue in the TPN group and increased in the TPN + GLP-2 group (all *p* < 0.001), whereas the control group showed normal intestinal tissue GSH level (Figure 5B).

### 3.4. Western Blot Analysis for PCNA, Cleaved Caspase-3 and GRP78 Protein

**Figure 6:**
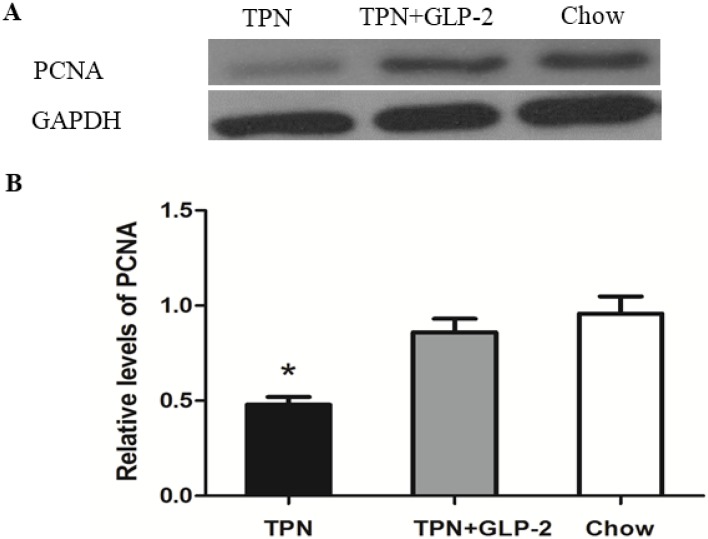
Western blot of the PCNA protein in ileum tissues. (**A**) Representative western blot results; (**B**) Relative density analysis of PCNA protein, * *p* < 0.05 *vs.* TPN + GLP-2 and control.

**Figure 7:**
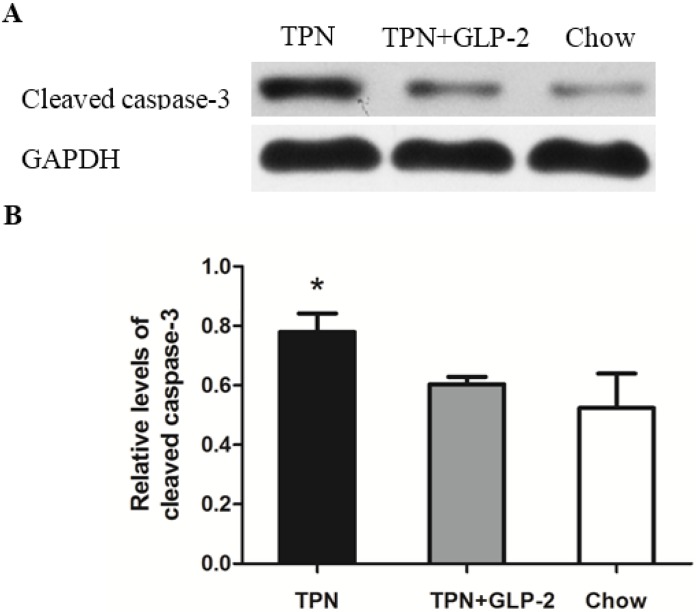
Western blot of the cleaved caspase-3 protein in ileum tissues. (**A**) Representative western blot results; (**B**) Relative density analysis of cleaved caspase-3 protein, * *p* < 0.05 *vs.* TPN + GLP-2 and control.

**Figure 8:**
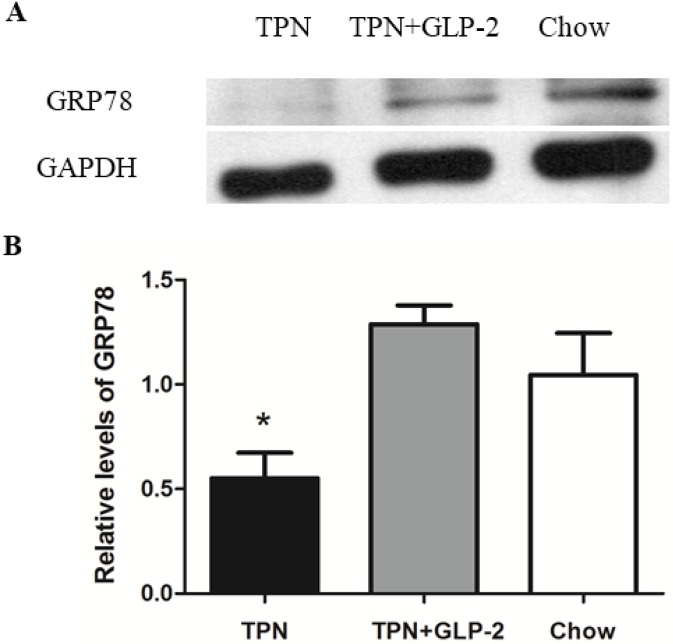
Western blot of the GRP78 protein in ileum tissues. (**A**) Representative western blot results; (**B**) Relative density analysis of GRP78 protein, * *p* < 0.05 *vs.* TPN + GLP-2 and control.

Expression of PCNA was significantly decreased in the TPN group compared to the control group (*p* < 0.001). However, TPN treatment with GLP-2 significantly improved the expression of PCNA protein (*p* = 0.001, Figure 6). Intestinal cleaved caspase-3 expression was 11.6% lower in the TPN + GLP-2 group compared with the TPN group (*p* = 0.032). There was no increase in cleaved caspase-3 in the control group, and that cleaved caspase-3 levels were 14.2% higher in the TPN group (*p* = 0.007, Figure 7). It is known that GRP78 plays an important role in protecting cells from oxidative injury [31]. Therefore, an assessment of inteasinalintestinal GPR78 protein was conducted. A significantly lower level of GRP78 was observed in the TPN group when compared to the control group (*p* = 0.006). Compared to TPN group, there were higher levels of GRP78 in the TPN + GLP-2 group (*p* = 0.001), but no difference compared to the control group (*p* = 0.086, Figure 8).

### 3.5. mRNA Expression of PCNA, GRP78

**Figure 9:**
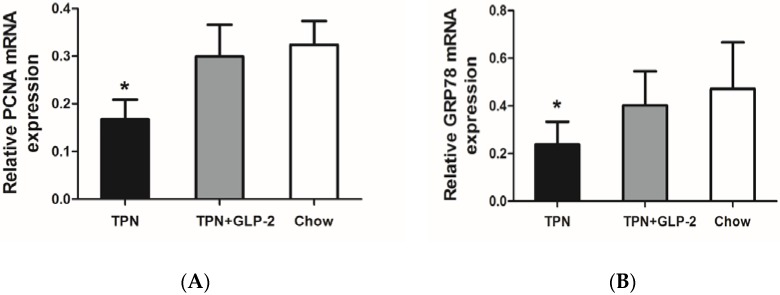
Effect of TPN, TPN + GLP-2 and control on mRNA expression. (**A**) Effects on PCNA mRNA, * *p* < 0.001 *vs.* TPN + GLP-2 and control; (**B**) Effects on GRP78 mRNA, * *p* < 0.05 *vs.* TPN + GLP-2 and control.

PCNA mRNA expression was significantly lower in the TPN group compared with the control group (*p* < 0.001), and this expression returned to nearly control levels in the TPN + GLP-2 group (*p* < 0.001 *vs.* TPN and *p* = 0.367 *vs.* control, Figure 9A). There was lower expression of GRP78 in the TPN group versus the control group (*p* = 0.006); however, levels in the TPN + GLP-2 group were not significantly different from those of the control group (*p* = 0.04 *vs.* TPN and *p* = 0.381 *vs.* control, Figure 9B).

## 4. Discussion

In our study, TPN administration led to significant alterations in intestinal morphology, including decreased villus length, crypt depth, and microvillus length compared to a control group, whereas GLP-2 supplementation significantly reduced these alterations associated with TPN. Notably, GLP-2 supplementation in the present TPN model significantly prevented TPN-associated intestinal atrophy through stimulating intestinal epithelial cell proliferation and inhibiting apoptosis. Another novel finding is that GLP-2 may promote epithelial cellular antioxidant capacity by upregulating GRP78 protein.

TPN administration is associated with many complications, including an increased risk of infection, loss of immune reactivity and epithelial barrier dysfunction, which are most likely due to intestinal mucosal atrophy [10]. It is well known that major contributing factors of TPN-associated intestinal mucosal atrophy are the reduced intestinal epithelial cell proliferation and increased apoptosis, embodied by a decline in the size of crypt/villus, which ultimately leads to a reduction in intestinal length [34,35,36]. Indeed, our study also strongly supports the hypothesis that TPN administration leads to a significant alteration in intestinal atrophy.

GLP-2 is one of the most important intestinotrophic hormones secreted by intestinal enteroendocrine L cells [13]. The precise biological role of GLP-2 has not been fully understood since it was discovered in 1996 [37], however, it is well known that GLP-2 is an important regulator of intestinal mucosa by stimulating epithelial growth [38]. Benjamin *et al.* first showed that GLP-2 and its analogue (h(Gly2)GLP-2) would improve the intestinal epithelial morphological changes in normal mice [39]. Therefore, one goal of our study was to investigate whether GLP-2 supplementation could have similar benefits in preventing TPN-associated atrophy. Indeed, in this present study, GLP-2-supplemented mice had longer small intestines, villi, and crypts compared to mice administered TPN alone. In addition, our electron microscopy studies demonstrated that the growth-enhancing properties of GLP-2 also applied to the intestinal epithelial microvilli in our mouse TPN model. Analogously, a study reported that GLP-2 revealed normal mucosa thickness and villus height of small intestine, whereas non-treated group maintained on TPN displaied villus shortening and thinning of mucosa [40]. In 2000, Burrin *et al.* found that GLP-2 increases intestinal growth in premature TPN-fed pigs [41]. Additionally, Martin and his colleagues also demonstrated that TPN plus GLP-2 treatment resulted in increased villus height, crypt cell proliferation, and intestinal mucosal surface area compared with the TPN alone rats [42]. Previous studies have shown that mechanisms of these positive morphological changes may account for the protective roles of GLP-2, such as stimulating crypt growth, inhibiting enterocyte apoptosis, and increasing absorption of nutrients [43,44]. Drucker *et al*. also demonstrated that GLP-2 administration increases small intestinal weight and villus growth by inducing a higher rate of proliferation in crypt cells [45]. In agreement with these results, the expression of PCNA protein, as well as its mRNA, was upregulated in the GLP-2-supplemented mice in the present study. This indicated that GLP-2 could reactivate enterocyte proliferation that has been negatively influenced by TPN. Additionally, Burrin *et al*. demonstrated that GLP-2 administration not only accelerated epithelial cell proliferation, but also reduced apoptosis markers in these enterocytes [46]. Here, we found that GLP-2 supplementation also reversed the increase in expression of cleaved caspased-3, suggesting that GLP-2 greatly suppresses apoptosis promoted by TPN. The effects of GLP-2-mediated suppression of apoptosis may be due to its trophic action [41]. Inhibition of apoptosis could reduce the breakdown of intestinal integrity, that is, that the intestine retains its integrity. In short, it is conceivable that the mechanism by which GLP-2 reduces intestinal mucosal atrophy may be due to regulation of enterocyte proliferation and apoptosis in a mouse model of TPN. Similarly, Chen *et al*. found that GLP-2 protects the intestinal barrier through epithelial cell proliferation and inhibition of apoptosis in rats with severe acute pancreatitis [47]. In our study, we found that GLP-2-supplemented mice had greater body weight than the mice administered TPN alone, especially at the sixth and seventh day, the difference was statistically significant. Previous animal studies had shown that GLP-2 or human analogue h(Gly^2^)GLP-2 significantly increased body weigth, weight of intestine or mucosal scraping [39,42]. In addition, because GLP-2 is an intestinal growth-promoting factor [13], so we consider that the increased body weigth in GLP-2-supplemented mice may was associated with the specific characteristics of GLP-2 on preventing intestinal atrophy. However, we need to acknowledge a limitation of our study is that we did not directly test the wet weight of intestine.

TPN administration can also cause a reactive oxygen species to be produced, and these peroxide contaminations could induce oxidative stress, leading to cell death [48,49]. Wesley *et al*. [50] found that free radicals were increased in neonates receiving TPN administration. In animal experiments, Eizaguirre *et al*. showed that TPN reduced tissue antioxidant capacity, which manifested in lower GSH levels [24]. GSH is a non-protein antioxidant and plays an important role in the protection against oxidative stress [51]. Similarly, T-SOD, one of the most extensively investigated enzymes, determines intracellular antioxidant capacity and plays a critical protective role in oxidative damage [52]. This study also provides evidence that TPN decreases antioxidant defense, and we found that GLP-2 treatment led to a significant increase in T-SOD activity and GSH level, which were otherwise downregulated in the TPN-fed mice. This result suggested that GLP-2 plays a crucial role in enhancing antioxidant capacity. GRP78 is an important molecular chaperone in the unfolded protein response, and overexpression of GRP78 has a marked effect in protecting cells from oxidative damage [53]. In the present study, it appears that GLP-2 supplementation affected intestinal GRP78 expression in the TPN-fed mice, as manifested by upregulation of GRP78 protein and mRNA expression. It should be noted that the effect of promoting antioxidant capacity of GLP-2 might be associated with the increased GRP78 expression. However, there are other factors involved in GRP78 expression in intestinal tissue, and further studies are needed to understand the mechanisms involved and the direct relationship between GRP78 and antioxidant capacity. Notably, overexpressed GRP78 was also associated with improved cell proliferation and reduced apoptosis [54]. We suspected that GLP-2 supplementation might promote GRP78 expression, which would lead to reduced enterocyte apoptosis. This suggests that overexpressed GRP78 may prevent intestinal mucosal atrophy. However, the mechanisms involved require further investigation.

## 5. Conclusions

In conclusion, the present study demonstrates that exogenous GLP-2 supplementation in mice receiving TPN partially prevented the TPN-associated intestinal mucus atrophy, and that GLP-2 stimulation of intestinal epithelial cell proliferation and suppression of apoptosis may be among the mechanisms involved. In addition, we found that GLP-2 treatment enhanced intestinal antioxidant capacity during TPN administration. GLP-2 plays an important intermediary role in increasing antioxidant capacity, and its mechanism may involve the overexpression of GRP78, although this requires further study. This study shows that the use of GLP-2 may be beneficial in preventing the development of clinical TPN-associated intestinal atrophy and oxidative damage.

## References

[1] Ekema G., Milianti S., Boroni G. (2009). Total parenteral nutrition in patients with short bowel syndrome. Minerva Pediatr..

[2] Triantafillidis J.K., Papalois A.E. (2014). The role of total parenteral nutrition in inflammatory bowel disease: Current aspects. Scand. J. Gastroenterol..

[3] Stanghellini V., Cogliandro R.F., de Giorgio R., Barbara G., Cremon C., Antonucci A., Fronzoni L., Cogliandro L., Naponelli V., Serra M. (2010). Natural history of intestinal failure induced by chronic idiopathic intestinal pseudo-obstruction. Transplant. Proc..

[4] Costakos D.T. (2006). Of lobsters, electronic medical records, and neonatal total parenteral nutrition. Pediatrics.

[5] Boullata J.I. (2012). Overview of the parenteral nutrition use process. JPEN J. Parenter. Enteral. Nutr..

[6] Feng Y., Teitelbaum D.H. (2012). Epidermal growth factor/TNF-αtransactivation modulates epithelial cell proliferation and apoptosis in a mouse model of parenteral nutrition. Am. J. Physiol. Gastrointest. Liver Physiol..

[7] Freeman J.J., Feng Y., Demehri F.R., Dempsey P.J., Teitelbaum D.H. (2015). TPN-associated intestinal epithelial cell atrophy is modulated by TLR4/EGF signaling pathways. FASEB J..

[8] Kudsk K.A., Croce M.A., Fabian T.C., Minard G., Tolley E.A., Poret H.A., Kuhl M.R., Brown R.O. (1992). Enteral *versus* parenteral feeding. Effects on septic morbidity after blunt and penetrating abdominal trauma. Ann. Surg..

[9] The Veterans Affairs Total Parenteral Nutrition Cooperative Study Group (1991). Perioperative total parenteral nutrition in surgical patients. N. Engl. J. Med..

[10] Feng Y., Ralls M.W., Xiao W., Miyasaka E., Herman R.S., Teitelbaum D.H. (2012). Loss of enteral nutrition in a mouse model results in intestinal epithelial barrier dysfunction. Ann. N. Y. Acad. Sci..

[11] Alverdy J.C., Aoys E., Moss G.S. (1988). Total parenteral nutrition promotes bacterial translocation from the gut. Surgery.

[12] Krishnan K., Arnone B., Buchman A. (2011). Intestinal growth factors: Potential use in the treatment of inflammatory bowel disease and their role in mucosal healing. Inflamm. Bowel. Dis..

[13] Estall J.L., Drucker D.J. (2006). Glucagon-like peptide-2. Annu. Rev. Nutr..

[14] Vegge A., Thymann T., Lund P., Stoll B., Bering S.B., Hartmann B., Jelsing J., Qvist N., Burrin D.G., Jeppesen P.B. (2013). Glucagon-like peptide-2 induces rapid digestive adaptation following intestinal resection in preterm neonates. Am. J. Physiol. Gastrointest. Liver Physiol..

[15] Yusta B., Holland D., Koehler J.A., Maziarz M., Estall J.L., Higgins R., Drucker D.J. (2009). ErbB signaling is required for the proliferative actions of GLP-2 in the murine gut. Gastroenterology.

[16] Burrin D.G., Stoll B., Guan X., Cui L., Chang X., Holst J.J. (2005). Glucagon-like peptide 2 dose-dependently activates intestinal cell survival and proliferation in neonatalpiglets. Endocrinology.

[17] Bremholm L., Hornum M., Andersen U.B., Hartmann B., Holst J.J., Jeppesen P.B. (2011). The effect of Glucagon-Like Peptide-2 on mesenteric blood flow and cardiac parameters in end-jejunostomy short bowel patients. Regul. Pept..

[18] Ivory C.P., Wallace L.E., McCafferty D.M., Sigalet D.L. (2008). Interleukin-10-independent anti-inflammatory actions of glucagon-like peptide 2. Am. J. Physiol. Gastrointest. Liver Physiol..

[19] Hadjiyanni I., Li K.K., Drucker D.J. (2009). Glucagon-like peptide-2 reduces intestinal permeability but does not modify the onset of type 1 diabetes in the nonobese diabetic mouse. Endocrinology.

[20] Arda-Pirincci P., Bolkent S. (2011). The role of glucagon-like peptide-2 on apoptosis, cell proliferation, and oxidant-antioxidant system at a mouse model of intestinal injury induced by tumor necrosis factor-alpha/actinomycin, D. Mol. Cell. Biochem..

[21] Arda-Pirincci P., Oztay F., Bayrak B.B., Yanardag R., Bolkent S. (2012). Teduglutide, a glucagon-like peptide 2 analogue: A novel protective agent with anti-apoptotic and anti-oxidant properties in mice with lung injury. Peptides.

[22] Topaloğlu N., Memi G., Kaner T., Deniz M., Şahin Ö., Güven M., Çoşar M. (2015). Does Glp-2 have a protective effect on cerebral ischemia/reperfusion model?. Turk. J. Med. Sci..

[23] Denno R., Rounds J.D., Faris R., Holejko L.B., Wilmore D.W. (1996). Glutamine-enriched total parenteral nutrition enhances plasma glutathione in the resting state. J. Surg. Res..

[24] Eizaguirre I., Aldámiz L., Aldazábal P., García Urkia N., Asensio A.B., Bachiller P., García Arenzana J.M., Ruiz J.L., Sanjurjo P., Perez Nanclares G. (2001). Tissue antioxidant capacity and bacterial translocation under total parenteral nutrition. Pediatr. Surg. Int..

[25] Cai W., Wu J., Hong L., Xu Y., Tang Q., Shi C. (2006). Oxidative injury and hepatocyte apoptosis in total parenteral nutrition-associated liver dysfunction. J. Pediatr. Surg..

[26] Jain A.K., Stoll B., Burrin D.G., Holst J.J., Moore D.D. (2012). Enteral bile acid treatment improves parenteral nutrition-related liver disease and intestinal mucosal atrophy in neonatal pigs. Am. J. Physiol. Gastrointest. Liver Physiol..

[27] Ivaylo S., Thomas H. (2009). PCNA on the crossroad of cancer. Biochem. Soc. Trans..

[28] Muskhelishvili L., Latendresse J.R., Kodell R.L., Henderson E.B. (2003). Evaluation of cell proliferation in rat tissues with BrdU, PCNA, Ki-67(MIB-5) immunohistochemistry and *in situ* hybridization for histone mRNA. J. Histochem. Cytochem..

[29] Porter A.G., Jänicke R.U. (1999). Emerging roles of caspase-3 in apoptosis. Cell. Death. Differ..

[30] Kozutsumi Y., Segal M., Normington K., Gething M.J., Sambrook J. (1988). The presence of malfolded proteins in the endoplasmic reticulum signals the induction of glucose-regulated proteins. Nature.

[31] He S., Yaung J., Kim Y.H., Barron E., Ryan S.J., Hinton D.R. (2008). Endoplasmic reticulum stress induced by oxidative stress in retinal pigment epithelial cells. Graefes. Arch. Clin. Exp. Ophthalmol..

[32] Yu Z., Luo H., Fu W., Mattson M.P. (1999). The endoplasmic reticulum stress-responsive protein GRP78 protects neurons against excitotoxicity and apoptosis: Suppression of oxidative stress and stabilization of calcium homeostasis. Exp. Neurol..

[33] Wan X., Bi J., Gao X., Tian F., Wang X., Li N., Li J. (2015). Partial enteral nutrition preserves elements of gut barrier function, including innate immunity, intestinal alkaline phosphatase (IAP) level, and intestinal microbiota in mice. Nutrients.

[34] Ekelund M., Kristensson E., Ekelund M., Ekblad E. (2007). Total parenteral nutrition causes circumferential intestinal atrophy, remodeling of the intestinal wall, and redistribution of eosinophils in the rat gastrointestinal tract. Dig. Dis. Sci..

[35] Wildhaber B., Lynn K., Yang H., Teitelbaum D.H. (2002). Total parenteral nutrition-induced apoptosis in mouse intestinal epithelium: Regulation by the Bcl-2 protein family. Pediatr. Surg. Int..

[36] Hua Y., Irfan K., Yongyi F., Forbush B., Bishop D.K., Antony P.A., Zhou H., Teitelbaum D.H. (2002). Interferon-gamma expression by intraepithelial lymphocytes results in a loss of epithelial barrier function in a mouse model of total parenteral nutrition. Ann. Surg..

[37] Rohallah M., Fatemeh N., Hossein N., Hassanzadeh G. (2011). Effect of different doses of GLP-2 (Teduglutide) on acute esophageal lesion due to acid-pepsin perfusion in male rats. Peptides.

[38] Wallis K., Walters J.R., Forbes A. (2007). Glucagon-like peptide-2-current applications and future directions. Aliment. Pharmacol. Ther..

[39] Benjamin M.A., McKay D.M., Yang P.C., Cameron H., Perdue M.H. (2000). Glucagon-like peptide-2 enhances intestinal epithelial barrier function of both transcellular andparacellular pathways in the mouse. Gut.

[40] Chance W.T., Foley-Nelson T., Thomas I., Balasubramaniam A. (1997). Prevention of parenteral nutrition-induced gut hypoplasia by coinfusion of glucagon-like peptide-2. Am. J. Physiol..

[41] Burrin D.G., Stoll B., Jiang R., Petersen Y., Elnif J., Buddington R.K., Schmidt M., Holst J.J., Hartmann B., Sangild P.T. (2000). GLP-2 stimulates intestinal growth in premature TPN-fed pigs by suppressing proteolysis and apoptosis. Am. J. Physiol. Gastrointest. Liver Physiol..

[42] Martin G.R., Wallace L.E., Sigalet D.L. (2004). Glucagon-like peptide-2 induces intestinal adaptation in parenterally fed rats with short bowel syndrome. Am. J. Physiol. Gastrointest. Liver Physiol..

[43] Tsai C.H., Hill M., Asa S.L., Brubaker P.L., Drucker D.J. (1997). Intestinal growth-promoting properties of glucagon-like peptide-2 in mice. Am. J. Physiol..

[44] Thulesen J. (2004). Glucagon-like peptide 2 (GLP-2), an intestinotrophic mediator. Curr. Protein. Pept. Sci..

[45] Drucker D.J., Erlich P., Asa S.L., Brubaker P.L. (1996). Induction of intestinal epithelial proliferation by glucagon-like peptide 2. Proc. Natl. Acad. Sci. USA.

[46] Burrin D.G., Stoll B., Guan X., Cui L., Chang X., Hadsell D. (2007). GLP-2 rapidly activates divergent intracellular signaling pathways involved in intestinal cell survival and proliferation in neonatal piglets. Am. J. Physiol. Endocrinol. Metab..

[47] Chen X., Zhao H.X., Fu X.S., Li C.P., Zhong X.L. (2012). Glucagonlike peptide 2 protects intestinal barrier in severe acute pancreatitis through regulatingintestinal epithelial cell proliferation and apoptosis. Pancreas.

[48] Szpetnar M., Matras P., Kiełczykowska M., Horecka A., Bartoszewska L., Pasternak K., Rudzki S. (2012). Antioxidants in patients receiving total parenteral nutrition after gastrointestinal cancer surgery. Cell. Biochem. Funct..

[49] Hasanoğlu A., Dalgiç N., Tümer L., Atalay Y., Cinasal G., Biberoğlu G., Bukan N., Aybar C. (2005). Free oxygen radical-induced lipid peroxidation and antioxidant in infants receiving total parenteral nutrition. Prostaglandins Leukot. Essent Fatty Acids.

[50] Wesley J.R., Coran A.G. (1992). Intravenous nutrition for the pediatric patient. Semin. Pediatr. Surg..

[51] Jia Z., Zhu H., Misra H.P., Li Y. (2008). Potent induction of total cellular GSH and NQO1 as well as mitochondrial GSH by 3H-1,2-dithiole-3-thione in SH-SY5Y neuroblastoma cells and primary human neurons: Protection against neurocytotoxicity elicited by dopamine, 6-hydroxydopamine, 4-hydroxy-2-nonenal, or hydrogen peroxide. Brain Res..

[52] Haddad J.J. (2004). Oxygen sensing and oxidant/redox-related pathways. Biochem. Biophys. Res. Commun..

[53] Suyama K., Watanabe M., Sakabe K., Otomo A., Okada Y., Terayama H., Imai T., Mochida J. (2014). GRP78 Suppresses Lipid Peroxidation and Promotes Cellular Antioxidant Levels in Glial Cells following Hydrogen Peroxide Exposure. PLoS ONE.

[54] Xiong Z., Jiang R., Li X., Liu Y., Guo F. (2015). Different Roles of GRP78 on Cell Proliferation and Apoptosis in Cartilage Development. Int. J. Mol. Sci..

